# Trend of prevalence and characteristics of preserved ratio impaired spirometry (PRISm): Nationwide population-based survey between 2010 and 2019

**DOI:** 10.1371/journal.pone.0307302

**Published:** 2024-07-23

**Authors:** Hyunji Choi, Chul-Ho Oak, Mann-Hong Jung, Tae-Won Jang, Sung-Jin Nam, Taemin Yoon

**Affiliations:** 1 Department of Laboratory Medicine, Kosin University Gospel Hospital, Kosin University College of Medicine, Busan, Republic of Korea; 2 Division of Pulmonology, Department of Internal Medicine, Kosin University Gospel Hospital, Kosin University College of Medicine, Busan, Republic of Korea; 3 Center for Future Medicine, Kosin University Gospel Hospital, Busan, Republic of Korea; Srebrnjak Children’s Hospital, CROATIA

## Abstract

**Purpose:**

This study aimed to evaluate the prevalence, trends, and factors of preserved ratio with impaired spirometry (PRISm) by using a nationally representative sample.

**Patients and methods:**

The datasets of the Korea National Health and Nutrition Examination Survey 2010–2019 were used: of total 32,949 participants aged ≥40 and no missing data on spirometry, 24,523 with normal, 4,623 with obstructive, and 3,803 with PRISm were identified. PRISm was defined as FEV_1_/FVC ≥70% and FEV_1_% of the predicted value (%pred) <80. PRISm-lower limit of normal (LLN) was defined when FEV_1_/FVC ≥LLN and FEV_1_ <LLN. The prevalence and trend of PRISm were estimated using the Joinpoint regression method. The trend was calculated for the periods 2010–2015 and 2017–2019, due to a change in spirometry device in June, 2016. A complex sample multivariable-adjusted regression model was used to identify factors associating PRISm.

**Results:**

Estimated average prevalence of PRISm was 10.4% (PRISm-LLN 11.1%). Joinpoint regression analyses found a relatively stable trend of PRISm for both fixed ratio and LLN. The multivariable-adjusted logistic regression model showed female sex, BMI ≥25 kg/m^2^, metabolic syndrome, hypertriglyceridemia, abdominal obesity, low HDL-choleterol, hypertension, and diabetes were associated with the increased probability of PRISm.

**Conclusion:**

Whenever a PRISm pattern is identified in a clinical context, it may be necessary to measure absolute lung volumes to investigate underlying physiological abnormalities and to identify factors that is modifiable.

## Introduction

Chronic obstructive pulmonary disease (COPD) is a chronic respiratory condition with persistent respiratory symptoms and airflow limitation [1]. COPD is still a growing public health burden with the third leading cause of death worldwide, attributing to 3.23 million deaths in 2019 [2]. There are substantial interest in identifying individuals at risk of progression to COPD [3], which have led to an introduction of early COPD, pre-COPD, or young COPD [3]. Recently, individuals with preserved ratio impaired spirometry (PRISm) have been highlighted as a potential risk of progression to COPD [4]. It is associated with increased respiratory symptoms, hospitalization, and mortality [5–7]. However, these patients are excluded from COPD clinical trials due to the spirometry definition of COPD since PRISm is defined as the FEV_1_/FVC ratio ≥0.7 but with reduced FEV_1_ (FEV_1_% of the predicted value [%pred] <80) [1].

Estimates of the prevalence of PRISm range from 5%–16.7% [4, 8–11]. PRISm is interchangeable with the term “restrictive spirometry pattern” with FVC %pred <80 in the presence of an FEV_1_/FVC ratio ≥0.7, which reflect the proportionate decrease in FEV_1_ and FVC levels when the ratio of FEV_1_/FVC is intact [12]. Prevalence of PRISm may vary according to the different ethnicities [13] and the definition that previous studies have adopted. PRISm is comprised of a heterogenous group with a wide range of comorbidities, body mass index (BMI), underlying lung conditions, and spirometry measurements [14]. Many epidemiologic studies have reported factors related to PRISm such as BMI, restrictive lung disease, and metabolic disorders like diabetes [10, 11, 14, 15]. The prevalence of those related factors has changed over time, however, the trend of prevalence of PRISm has not been assessed thoroughly. An epidemiologic study from the United States showed the prevalence of restrictive spirometry pattern decreased from 7.2% in 1988–1994 to 5.4% in 2007–2010 [16], but recent trend using the current definition of PRISm has not been investigated. Therefore, investigating the trend of prevalence of PRISm and its related factors could provide insights for public health in terms of early detection and intervention, policy development, or evaluation of public health programs. In addition, identification of related factors which is modifiable could inform clinicians to encourage their patients towards lifestyle modification.

In this regard, the present study aims to examine the prevalence of PRISm and its secular trend, and clinical characteristics related to PRISm in Korean population using nationwide representative sample.

## Material and methods

### Datasets and study participants

The present study used data from 2010–2019 Korea National Health and Nutrition Examination Survey (KNHANES). The survey was conducted annually by the Korean Centers for Disease Control and Prevention. The KNHANES collects nationally representative data of non-institutionalized Korean citizens. The data comprises health interviews, health examinations, and nutrition surveys. Each survey year included a new sample of randomly selected individuals. Detailed procedures of the KNHANES have been described previously [17].

We collected the data of KNHANES from 2010–2019, where of total 103,465 individuals, 80,860 (78.2%) responded the survey. Because pulmonary function testing (PFT) was only performed in adults aged ≥40 years, participants <40 were excluded (n = 47,911). Of the remaining 32,949 individuals, 24,523 with normal, 4,623 with obstructive, and 3,803 with PRISm spirometry pattern were remained and included in the analysis.

### Spirometry and PRISm

PFT was performed using dry rolling seal spirometers (Model 2130; SensorMedics, Yorba Linda, CA, USA) until 2016 and Vyntus Spiro (CareFusion, San Diego, CA, USA) after 2016. The device for PFT was changed in June, 2016. Calibration and quality control followed the standardization criteria of the American Thoracic Society and European Respiratory Society [18]. Forced expiratory volume in 1 second (FEV_1_, Liters), forced vital capacity (FVC, Liters), and the ratio of FEV_1_/FVC (%) were obtained from the prebronchodilator test. Postbronchodilator testing was not performed in the KNHANES. The values of the FEV_1_%pred and FVC %pred were using the reference equation from a representative sample of Koreans [19]. In addition, because of concerns about the spread of SARS-CoV-2, pulmonary function test was not performed in 2020 and 2021.

Normal pattern of spirometry was defined when pre-bronchodilator is both FEV_1_/FVC% ≥70 and FEV_1_%pred ≥80. Obstructive pattern was defined when FEV_1_/FVC% <70. PRISm pattern was defined the value is FEV_1_/FVC ≥70% and FEV_1_%pred <80 [4]. In addition to the fixed ratio of 70%, lower limit of normal (LLN) thresholds for FEV_1_/FVC and FEV_1_ based on Global Lung Function Initiative (GLI) reference equations were adopted [6, 20, 21]: normal-LLN pattern of spirometry was defined as FEV_1_/FVC ≥LLN and FEV_1_ ≥LLN, obstructive-LLN was defined as FEV_1_/FVC <LLN, and PRISm-LLN was defined as FEV_1_/FVC ≥LLN and FEV_1_ <LLN and. Concordance rate between fixed ratio and LLN for the classification of normal pattern, obstructive pattern, and PRISm was 83.1% (**S1 Table**)

### Variables

Demographic variables include age, sex, and residence. Smoking status was categorized into never, former, and current smokers based on the National Health Interview Survey of the United States. Current smokers were defined as individuals who smoked more than 100 cigarettes in their lifetime and who currently smoked. Former smokers were defined as individuals who smoked more than 100 cigarettes in their lifetime but had stopped smoking for more than 1 year. Body mass index (kg/m^2^) was classified into <23 (underweight or normal), 23 –<25 (pre-obese), and ≥25 (obese) based on the Korean guideline of the obesity [22].

### Statistical analysis

The KNHANES was designed to represent non-institutionalized South Korean citizens by a stratified multistage probability sampling method was employed. Therefore, all statistical analyses conducted in this study considered sampling weights, stratification, and clustering of the KNHANES data [17].

The Age-standardized prevalence of PRISm was shown with the estimated percentage. Standardization was done based on the 2010 population census data for all South Koreans. Factors related to PRISm were analyzed using complex sample multivariable-adjusted logistic regression method to estimate weighted odds ratio (OR) and 95% confidence interval (CI) of PRISm. Age, sex, residence, smoking status, and BMI were adjusted for the model.

A joinpoint regression analysis was performed to identify any changes in the trend of prevalence of PRISm with the use of Joinpoint Regression Program version 5.0.2 (Statistical Research and Applications Branch, National Cancer Institute, USA). Because the device for PFT was changed in June, 2016, we separated the whole periods into 2010–2015 and 2017–2019 to calculate annual percent change (APC). Number of joinpoint year was determined based on the recommendation using grid search method.

All statistical analyses for the complex sample survey were performed using SPSS (version 24 for Windows, Chicago, USA). For all analyses, *p-*value < 0.05 was considered statistically significant.

### Ethical statement

The study protocol was approved by the Institutional Review Board of Kosin University Gospel Hospital (no. 2024-03-024). The study was conducted in accordance with the Declaration of Helsinki. All procedures were performed in accordance with relevant guidelines and regulations. Patient consent was waived due to the retrospective nature of this study.

## Results

The estimated population size during the period 2010–2019 was 72,949,077 for normal spirometry pattern, 3,138,731 for obstructive pattern, and 8,981,827 for PRISm, respectively (**Table 1**). Individuals with PRISm were younger, had higher BMI, lower FEV_1_, FEV_1_%pred, FVC, FVC %pred, and FEV_1_/FVC compared to those with a normal spirometry pattern. Compared to individuals with an obstructive pattern, those with PRISm were younger, more likely to be female, lived in town, were never smokers, and had higher BMI, as well as lower FVC and FVC %pred.

**Table 1:** Characteristics of study participants aged ≥40 according to fixed ratio.

Year	Normal	Obstructive	PRISm	*p*
**Population size**	72,949,077	3,138,731	8,981,827	
**Age**				<0.001*,†, ‡
40–49	37.3 (0.5)	9.2 (0.6)	33.7 (1.1)	
50–59	31.6 (0.4)	22.0 (0.8)	34.9 (1.0)	
60–69	18.5 (0.3)	30.8 (0.8)	19.3 (0.8)	
≥70	12.6 (0.3)	38.0 (0.9)	12.1 (0.7)	
**Sex**				<0.001*,‡
Male	43.4 (0.3)	74.0 (0.8)	50.1 (1.0)	
Female	56.6 (0.3)	26.0 (0.8)	49.9 (1.0)	
**Residence**				<0.001*,‡
Town	81.3 (0.9)	74.9 (1.4)	81.5 (1.2)	
Rural	18.7 (0.9)	25.1 (1.4)	18.5 (1.2)	
**Smoking**				<0.001*,‡
Never	61.8 (0.4)	31.5 (0.8)	55.2 (1.1)	
Former	20.5 (0.3)	38.0 (0.9)	23.1 (0.9)	
Current	17.7 (0.3)	30.5 (0.8)	21.7 (0.9)	
**BMI** (kg/m^2^)				<0.001*,†, ‡
<23	36.1 (0.4)	41.4 (0.9)	32.3 (1.0)	
23–24.9	26.6 (0.3)	27.3 (0.8)	25.1 (0.9)	
≥25	37.3 (0.4)	31.3 (0.9)	42.6 (1.1)	
**FEV**_**1**_ **(L)**	2.84 (0.01)	2.28 (0.01)	2.27 (0.01)	<0.001*,†
**FEV**_**1**_ **%pred**	95.6 (0.1)	77.5 (0.3)	74.2 (0.1)	<0.001*,†
**FVC (L)**	3.58 (0.01)	3.58 (0.01)	2.96 (0.02)	<0.001†, ‡
**FVC %pred**	94.2 (0.1)	89.1 (0.3)	75.6 (0.2)	<0.001*,†, ‡
**FEV**_**1**_ **/FVC%**	79.7 (0.0)	63.5 (0.1)	76.9 (0.1)	<0.001*,†, ‡

Data are presented as mean (standard error) for continuous variables and percentage (standard error) for categorical variables.

* p <0.017 normal vs obstructive

† p <0.017 normal vs PRISm, and

‡ p <0.017 obstructive vs PRISm.

PRISm = preserved ratio impaired spirometry; BMI = body mass index; FEV_1_ = forced expiratory volume in one second; FVC = forced vital capacity.

Age-standardized prevalence of PRISm based on fixed ratio was shown in **Table 2**. Weighted estimate of average prevalence during 2010–2019 in adults aged ≥40 was 10.4%. A joinpoint regression analysis revealed stable trend during both 2010–2015 and 2017–2019. The prevalence among individuals aged 60–69 decreased during both periods. Among individuals aged 40–49, during 2017–2019 the trend of prevalence increased in males while it decreased in females. The estimates of the prevalence based on LLN were presented in **Table 3**. Average prevalence of PRISm-LLN during 2010–2019 was 11.4%. The trend of prevalence was stable during 2010–2015 and slight increase during 2017–2019.

**Table 2:** Age-standardized prevalence (%) of PRISm.

	All	2010	2011	2012	2013	2014	2015	APC1	2017	2018	2019	APC2
**Total**	**10.4**	7.6	8.5	8.9	7.6	7.7	7.5	-1.5	13.9	15.7	14.0	0.4
40–49	**10.6**	7.6	9.0	10.4	8.2	8.1	9.3	1.3	17.7	18.0	17.7	0.0
50–59	**11.8**	8.0	10.1	9.1	8.5	9.5	7.0	-2.6	14.3	19.3	15.8	5.1
60–69	**9.9**	8.8	8.6	8.9	6.7	6.2	5.7	-9.4*	10.9	10.3	10.2	-3.3*
≥70	**7.9**	5.3	4.5	5.0	5.8	4.8	7.4	5.9	9.6	11.9	9.6	0.0
**Male**	**10.8**	8.2	10.0	6.8	8.1	8.0	7.1	-3.4	14.9	18.1	15.6	2.3
40–49	**11.7**	8.9	10.8	8.9	8.7	8.0	10.2	-0.7	20.9	21.7	21.6	1.7*
50–59	**12.1**	8.5	12.0	6.2	8.5	8.9	6.2	-6.0	15.1	22.4	16.1	3.3
60–69	**10.1**	9.4	10.6	7.0	7.7	8.5	4.6	-11.2	11.0	12.4	11.0	0.0
≥70	**6.9**	3.3	2.1	2.4	6.1	5.2	5.1	18.2	6.5	9.4	9.2	19.0*
**Female**	**10.0**	7.0	7.2	10.8	7.2	7.3	7.9	0.7	13.0	13.5	12.5	-1.9
40–49	**9.4**	6.3	7.2	12.1	7.6	8.2	8.4	4.0	14.3	14.3	13.7	-2.1*
50–59	**11.6**	7.5	8.1	12.0	8.4	10.1	7.8	1.4	13.5	16.2	15.5	7.2
60–69	**9.8**	8.2	6.9	10.7	6.0	4.7	6.3	-8.3	10.6	8.7	9.5	-5.3
≥70	**8.7**	6.5	6.0	6.7	5.3	4.0	9.7	1.6	12.9	14.1	9.7	-13.3

*APC is significantly different from 0.

APC1 was calculated between 2010 and 2015 and APC2 was calculated between 2017 and 2019.

Age-standardized prevalence was calculated using the 2010 population census data for all South Koreans.

PRISm = preserved ratio impaired spirometry; APC = annual percentage change.

**Table 3:** Age-standardized prevalence (%) of PRISm based on the cut-off values using LLN (5^th^ percentile).

	All	2010	2011	2012	2013	2014	2015	APC1	2017	2018	2019	APC2
**Total**	**11.1**	9.0	9.3	10.0	9.8	8.3	9.9	0.3	14.1	14.2	14.5	1.4*
40–49	**14.1**	10.1	11.0	12.9	13.5	9.7	14.8	4.6	20.6	20.1	21.1	1.2
50–59	**11.2**	9.6	10.2	9.7	9.3	10.2	8.1	-2.5	12.8	15.2	15.2	8.9*
60–69	**7.8**	7.0	7.2	8.8	6.7	5.0	6.0	-5.9	9.8	7.9	7.7	-11.4*
≥70	**8.7**	7.5	6.4	5.9	6.1	5.6	8.5	0.7	9.6	9.6	11.0	7.0*
**Male**	**3.9**	2.6	2.5	2.7	2.9	3.1	2.7	2.6	4.8	6.3	6.5	16.4*
40–49	**4.9**	3.2	3.2	4.3	4.2	2.7	5.0	5.0	7.3	9.9	7.7	2.7
50–59	**3.8**	2.8	3.6	2.1	2.5	4.4	1.4	-7.4	4.1	7.4	6.8	28.8
60–69	**2.5**	2.8	0.9	1.6	1.9	1.6	1.2	-6.5	2.6	2.5	4.8	35.9
≥70	**2.9**	0.5	0.3	1.1	1.5	2.8	2.3	51.0*	3.7	1.9	5.6	23.0
**Female**	**17.9**	14.9	15.6	16.8	16.1	13.0	16.5	-0.2	22.7	21.6	22.1	-1.3
40–49	**23.6**	17.2	19.2	21.8	23.0	16.9	24.8	4.4	34.3	30.7	35.1	1.2
50–59	**18.6**	16.5	16.8	17.3	16.2	16.1	14.8	-2.1	21.6	23.0	23.5	4.3*
60–69	**11.5**	10.8	12.7	15.3	9.8	6.9	9.0	-8.7	13.7	11.7	9.9	-15.0*
≥70	**13.6**	11.8	10.2	8.9	10.1	8.3	14.6	1.7	16.4	17.0	16.0	-1.2

*APC is significantly different from 0.

APC1 was calculated between 2010 and 2015 and APC2 was calculated between 2017 and 2019.

Age-standardized prevalence was calculated using the 2010 population census data for all South Koreans.

PRISm = preserved ratio impaired spirometry; APC = annual percentage change.

In a multivariable-adjusted model, older individuals exhibited significantly lower OR for having PRISm as well as PRISm-LLN compared to younger individuals aged 40–49 (**Table 4**). Model based on fixed ratio showed that high BMI (≥25 kg/m^2^), metabolic syndrome, and its components (hypertriglyceridemia, abdominal obesity, low HDL-cholesterol, hypertension, and ***diabetes*** mellitus) were associated with significantly higher OR of PRISm. In terms of PRISm-LLN, results were consistent with more prominent association with sex.

**Table 4:** Multivariable-adjusted model for factors associating PRISm compared to normal spirometry pattern based on the fixed ratio of 0.7 or LLN (5^th^ percentile).

	Fixed ratio		LLN ^a)^	
	OR (95% CI)	*p*	OR (95% CI)	*p*
**Age**		**<0.001**		**<0.001**
40–49	*Reference*		*Reference*	
50–59	1.10 (0.98–1.24)		0.83 (0.75–0.92)	
60–69	0.87 (0.77–0.99)		0.55 (0.49–0.62)	
≥70	0.68 (0.58–0.79)		0.52 (0.45–0.61)	
**Sex**		0.746		**<0.001**
Male	*Reference*		*Reference*	
Female	1.02 (0.89–1.18)		5.22 (4.40–6.20)	
**Residence**		0.328		0.628
Urban	*Reference*		*Reference*	
Rural	0.95 (0.85–1.07)		1.03 (0.92–1.15)	
**Smoking**		0.338		0.741
Never	*Reference*		*Reference*	
Former	1.01 (0.87–1.19)		1.08 (0.90–1.30)	
Current	1.08 (0.92–1.26)		0.96 (0.79–1.16)	
**BMI** (kg/m^2^)		**0.034**		**<0.001**
<23	*Reference*		*Reference*	
23–24.9	1.01 (0.90–1.14)		1.07 (0.95–1.20)	
≥25	1.13 (1.01–1.26)		1.31 (1.18–1.45)	
**Metabolic disorders** ^**b)**^				
Metabolic syndrome	1.43 (1.29–1.59)	**<0.001**	1.28 (1.15–1.43)	**<0.001**
Hypertriglyceridemia	1.25 (1.14–1.37)	**<0.001**	1.18 (1.07–1.30)	**0.001**
Abdominal obesity	1.48 (1.31–1.68)	**<0.001**	1.31 (1.16–1.49)	**<0.001**
Low HDL-cholesterol	1.10 (1.01–1.21)	**0.039**	0.98 (0.89–1.07)	0.633
Hypertension	1.27 (1.16–1.39)	**<0.001**	1.15 (1.05–1.27)	**0.003**
Diabetes mellitus	1.33 (1.21–1.45)	**<0.001**	1.22 (1.11–1.34)	**<0.001**

Bold denotes *p* <0.05.

a) Normal = FEV_1_/FVC ≥ LLN and FEV_1_ ≥LLN; PRISm = FEV_1_/FVC ≥ LLN and FEV_1_ < LLN.

b) Estimates for metabolic syndrome and its individual components were calculated individually with adjustment for age, sex, residence, smoking, and BMI.

PRISm = preserved ratio impaired spirometry; LLN = lower limit of normal; BMI = body mass index; FVC = forced vital capacity; HDL = high density lipoprotein; OR = odds ratio; CI = confidence interval.

## Discussion

For this study, a large nationally representative sample of a period from 2010 to 2019 with an estimated population size of more than 22,000,000 individuals every year was utilized. we investigated the trend of prevalence and clinical characteristics of individuals with PRISm. The average prevalence in all studied participants was 10.4% (11.1% for PRISm-LLN). During the period, the trend of prevalence of PRISm and PRISm-LLN was relatively stable. Factors related to an increase in OR of PRISm were female sex (only for PRISm-LLN), high BMI (≥25 kg/m^2^), metabolic syndrome, hypertriglyceridemia, abdominal obesity, low HDL-cholesterol (not for PRISm-LLN), hypertension, and diabetes. Given that the results from this study were not based on absolute lung volumes and the related factors were mostly extra-pulmonary, it would be necessary to investigate underlying physiological abnormalities using total lung capacity and to identify modifiable extra-pulmonary conditions in real clinical practice.

Our study expanded previous epidemiologic studies that reported the prevalence of PRISm at a certain time [4, 6, 9, 14, 23, 24] showing a relatively stable trend of PRISm. A study decades ago from the NHANES of the United States [16] found decreasing trend from 1998–1994 to 2007–2010, implicating the decreasing trend of obesity. However, when we expanded this finding for more recent period, 2010–2019, the trend was not significantly changed recently. The percentages of adjusted variables in this study were also relatively constant across the study period (**S2 Table**). Given certain proportion of PRISm could progress to clinical COPD [25] and could be relate to cardiovascular disease [26] as well as mortality [4, 6, 23, 24, 27], this proportion of PRISm could contribute to significant public health burdens in the future. Moreover, complexity in the mechanism of PRISm could make decision for treatment challenging [28].

One of main findings of our study is about the associating factors with PRISm: younger age, female sex (only for PRISm-LLN), high BMI (≥25 kg/m^2^), and metabolic syndrome and its components. This finding corroborates previous reports. In terms of age, compared to individuals aged 40–49, the probability of having PRISm decreased as age increases. This finding would be related to the increasing prevalence of clinical COPD in elderly individuals [29]. Indeed, individuals with obstructive pattern were significantly older than those with PRISm (**Table 1**). Data from the COPDGene cohort showed female sex as a significant predictor of PRISm, where the probability of PRISm in male was 30% lower than that of female [14]. It has been widely described that individuals with PRISm have higher BMI than normal spirometry and obstructive spirometry [4, 6, 14, 15, 30]. In addition, metabolic syndrome and its components have been consistently associated with the restrictive spirometry pattern [31]. The presence of metabolic syndrome in individuals with PRISm could additionally increase the risk of cardiovascular disease, respiratory disease, and death for any cause [32]. A recent genome wide association study of PRISm also supports the possible relationship with metabolic disorders showing a strong genetic correlation with type 2 diabetes and PRISm [33].

Although we observed some factors associated with PRISm, this study was not based on absolute lung function measurement. Additionally, the related factors of PRISm were mostly extra-pulmonary. Therefore, understanding underlying physiological mechanisms related to PRISm would be of importance. PRISm refers to a group of lung function characterized by a decrease in FEV_1_ while maintaining a relatively constant FEV_1_/FVC. This phenomenon has been classified as restrictive pattern [12, 27], GOLD-unclassifiable [34], and non-specific pattern [35]. One pathophysiologic study in China found computed tomography (CT)-measured total lung capacity (TLC) was significantly lower in individuals with PRISm compared to healthy controls and patients with COPD [36]. A Canadian population-based study indicated PRISm was associated with lower exercise capacity and higher exertional dyspnea [37]. Furthermore, it has been suggested that there is a relationship between small airway disease and PRISm. Individuals with PRISm exhibited more severe small airway disease than those with a normal pattern [36, 38]. Given the proportional decrease in both FEV_1_ and FVC in individuals with PRISm, abnormal lung growth leading to low maximally attained lung function [30] might be related to the emergence of PRISm.

Our study has several limitations. First, the KNHANES is a cross-sectional study. This study does not consider for temporality. The causal relationships between factors and PRISm are not clear, and further studies are necessary. Second, post-bronchodilator test was not performed in the KNHAES. While post-bronchodilator spirometry may not be necessary for the classification of PRISm [24], its inclusion could reduce the proportion of individuals identified as having PRISm, and differences may exist between pre- and post-bronchodilator spirometry [39]. Third, this study lacks additional lung function measurements to investigate the physiological abnormalities underlying PRISm. For example, further studies using total lung capacity could more accurately classify individuals with PRISm [36]. Fourth, this study could not evaluate the longitudinal impact of PRISm. Natural trajectory and clinical outcomes of PRISm in this population should be further investigated. Fifth, because this study analyzed the population-based data of South Korea, the generalizability across other countries or ethnicities might not be ascertained. Finally, considering that the KNHANES is a complex sample survey, one should consider the possibility of selection bias. Unequal selection and the potential for biased population estimates may arise in the context of a complex sample survey [40]. Therefore, to guarantee accurate estimates and standard errors, this study employed complex samples analyses in SPSS for all statistical procedures.

## Conclusion

With the use of large nationally representative sample, we assessed the prevalence and 10-year trajectory of PRISm. There observed a relatively stable trend of prevalence of PRISm during 2010–2019. Identified factors linked to PRISm include younger age, female sex (only for PRISm-LLN), high BMI (≥25 kg/m^2^), metabolic syndrome, hypertriglyceridemia, abdominal obesity, low HDL-cholesterol (not for PRISm-LLN), hypertension, and diabetes. Whenever a PRISm pattern is identified in a clinical context, it is necessary to measure absolute lung volumes to investigate underlying physiological abnormalities. Additionally, since the related factors primarily relate to extra-pulmonary aspects, it is important to identify modifiable extra-pulmonary conditions.

## Supporting information

S1 Table Difference of the prevalence between the fixed ratio and LLN. (DOCX)

S2 Table Unadjusted prevalence (%) of variables in adults aged ≥40. (DOCX)
