# Supplementary material for: Trend of prevalence and characteristics of preserved ratio impaired spirometry (PRISm): Nationwide population-based survey between 2010 and 2019
Source: PLoS One. 2024 Jul 23;19(7):e0307302. doi: 10.1371/journal.pone.0307302 (PMC11265705; doi:10.1371/journal.pone.0307302)
Supplement: S1 Table — (DOCX) [file pone.0307302.s001.docx]

**S1 Table.** Difference of the prevalence between the fixed ratio and LLN

| **Fixed ratio ^a)^** |  | **LLN ^b)^** | | | |
| --- | --- | --- | --- | --- | --- |
|  |  | **Normal** | **Obstructive** | **PRISm** | **Total** |
| **Normal** | n. | 22058 | 342 | 2123 | 24523 |
|  | (%) | **66.9** | 1.0 | 6.4 | 74.4 |
| **Obstructive** | n. | 864 | 3575 | 184 | 4623 |
|  | (%) | 2.6 | **10.9** | 0.6 | 14.0 |
| **PRISm** | n. | 1891 | 172 | 1740 | 3803 |
|  | (%) | 5.7 | 0.5 | **5.3** | 11.5 |
| **Total** | n. | 24813 | 4089 | 4047 | 32949 |
|  | (%) | 75.3 | 12.4 | 12.3 | 100.0 |

a) Based on fixed ratio, normal spirometry pattern was defined when FEV_1_/FVC ≥70 and FEV_1_ %pred ≥80; obstructive spirometry pattern when FEV_1_/FVC% <70; and PRISm when FEV_1_/FVC% ≥70 and FEV_1_ %pred <80.

b) Based on LLN, normal spirometry pattern was defined when FEV_1_/FVC ≥ LLN and FEV_1_ ≥LLN; obstructive spirometry pattern when FEV_1_/FVC < LLN; and PRISm when FEV_1_/FVC ≥ LLN and FEV_1_ < LLN.

Bold denotes the percentage of concordance between the methods for classification.

LLN = lower limit of normal; PRISm = preserved ratio impaired spirometry; FEV_1_ = forced expiratory volume in one second; FVC = forced vital capacity; %pred = % of the predicted value.
