# Supplementary material for: Trend of prevalence and characteristics of preserved ratio impaired spirometry (PRISm): Nationwide population-based survey between 2010 and 2019
Source: PLoS One. 2024 Jul 23;19(7):e0307302. doi: 10.1371/journal.pone.0307302 (PMC11265705; doi:10.1371/journal.pone.0307302)
Supplement: S2 Table — (DOCX) [file pone.0307302.s002.docx]

**S2 Table**. Unadjusted prevalence (%) of variables in adults aged ≥40

|  | **All** | **2010** | **2011** | **2012** | **2013** | **2014** | **2015** | **2016** | **2017** | **2018** | **2019** | **APC** |
| --- | --- | --- | --- | --- | --- | --- | --- | --- | --- | --- | --- | --- |
|  |  |  |  |  |  |  |  |  |  |  |  |  |
| **Age** |  |  |  |  |  |  |  |  |  |  |  |  |
| 40-49 | 33.2 | 37.0 | 36.3 | 35.3 | 34.4 | 33.5 | 32.6 | 32.5 | 31.5 | 30.7 | 29.5 | -2.4* |
| 50-59 | 30.7 | 29.7 | 30.0 | 30.7 | 31.1 | 31.3 | 31.3 | 31.1 | 30.9 | 30.5 | 30.4 | 0.2 |
| 60-69 | 20.2 | 17.7 | 17.5 | 17.5 | 21.4 | 21.2 | 21.8 | 19.1 | 21.1 | 21.8 | 22.3 | 2.6* |
| ≥70 | 15.9 | 15.7 | 16.2 | 16.6 | 13.1 | 14.0 | 14.3 | 17.3 | 16.5 | 17.0 | 17.8 | 1.4 |
| **Residence** |  |  |  |  |  |  |  |  |  |  |  |  |
| Rural | 28.2 | 24.3 | 23.2 | 20.2 | 18.9 | 18.9 | 16.2 | 15.6 | 14.3 | 18.5 | 19.6 | -3.6 |
| Urban | 80.4 | 71.8 | 75.7 | 76.8 | 79.8 | 81.1 | 81.1 | 83.8 | 84.4 | 85.7 | 81.5 | 1.6* |
| **Smoking** |  |  |  |  |  |  |  |  |  |  |  |  |
| Never | 57.1 | 55.4 | 55.8 | 56.7 | 58.9 | 58.5 | 57.6 | 57.5 | 57.7 | 56.5 | 56.1 | 0.1 |
| Former | 23.1 | 21.1 | 22.1 | 21.7 | 21.0 | 20.3 | 24.4 | 23.1 | 24.9 | 24.5 | 26.3 | 2.4* |
| Current | 19.8 | 23.5 | 22.1 | 21.5 | 20.0 | 21.3 | 18.0 | 19.4 | 17.4 | 19.0 | 17.7 | -2.9* |
| **BMI (kg/m^2^)** |  |  |  |  |  |  |  |  |  |  |  |  |
| <23 | 36.4 | 36.1 | 33.9 | 34.3 | 36.4 | 38.8 | 35.4 | 35.2 | 36.7 | 38.2 | 38.7 | 2.8* |
| 23-24.9 | 26.5 | 26.1 | 28.1 | 26.5 | 27.6 | 25.6 | 26.9 | 25.7 | 27.1 | 26.0 | 25.8 | -0.4 |
| ≥25 | 37.0 | 37.8 | 38.0 | 39.3 | 35.9 | 35.6 | 37.7 | 39.1 | 36.2 | 35.8 | 35.5 | -0.6 |
| Metabolic syndrome | 34.3 | 35.9 | 35.8 | 33.7 | 32.6 | 31.5 | 35.8 | 36.8 | 32.2 | 32.9 | 36.0 | -0.2 |
| Hypertriglyceridemia | 34.3 | 35.3 | 35.8 | 37.4 | 35.9 | 34.0 | 34.2 | 35.1 | 31.9 | 32.2 | 32.4 | -1.4* |
| Abdominal obesity | 31.9 | 31.3 | 34.4 | 29.7 | 25.8 | 28.1 | 34.1 | 34.8 | 30.0 | 31.1 | 39.2 | 1.5 |
| Low HDL-cholesterol | 36.6 | 47.2 | 40.2 | 39.7 | 37.5 | 35.9 | 37.0 | 36.6 | 33.7 | 33.7 | 28.4 | -4.0* |
| Hypertension | 48.9 | 53.6 | 50.8 | 50.1 | 47.2 | 44.8 | 47.9 | 50.5 | 47.1 | 48.7 | 49.6 | -0.6 |
| Diabetes | 42.1 | 36.3 | 34.9 | 39.6 | 42.6 | 42.1 | 44.5 | 45.1 | 43.6 | 44.0 | 46.1 | 2.7* |

*APC is significantly different from 0.

APC = annual percentage change; BMI = body mass index; HDL = high density lipoprotein.
